# Time-dynamic perspectives on cancer therapy–related cardiotoxicity: a systematic review and time-course evidence synthesis for multibiomarker monitoring

**DOI:** 10.3389/fcvm.2026.1886989

**Published:** 2026-08-19

**Authors:** Chang Su, Siyuan Wan, Menghan Shen, Shun Zhang, Meijun Jia, Yili Yao, Yabin Gong, Qian Ba, Chengzeng Yao

**Affiliations:** 1 Department of Cardiology, Shuguang Hospital Affiliated to Shanghai University of Traditional Chinese Medicine, Shanghai, China; 2 Yueyang Hospital of Integrated Traditional Chinese and Western Medicine Affiliated to Shanghai University of Traditional Chinese Medicine, Shanghai, China; 3 Science and Technology Innovation Center, Shanghai Municipal Hospital of Traditional Chinese Medicine, Shanghai University of Traditional Chinese Medicine, Shanghai, China

**Keywords:** anthracyclines, biomarkers, cardio-oncology, cardiotoxicity monitoring, evidence mapping, HER2 inhibitors, time-course evidence synthesis

## Abstract

**Objective:**

Current monitoring of cancer therapy–related cardiac dysfunction relies largely on threshold-based interpretation at isolated time points, yet its temporal evolution remains insufficiently characterized. This review aims to map longitudinal monitoring evidence across treatments, biomarkers, and time windows; reconstruct trajectories of key biochemical and imaging markers in evidence-rich settings; and explore temporal patterns between biochemical injury signals and imaging-detected functional changes.

**Methods:**

PubMed and Embase were searched from inception to 1 October 2025. A three-dimensional evidence map was constructed across treatment modality, monitoring marker, and time window using a custom four-level evidence grading system based on data completeness and extractability. Quantitative synthesis was restricted to cohorts providing extractable longitudinal absolute values at mappable follow-up times in non-outcome-driven designs. Standardized trajectories of hs-cTnI/T, NT-proBNP, left ventricular ejection fraction (LVEF), and global longitudinal strain (GLS) were aggregated within predefined time windows.

**Results:**

Forty-six independent cohorts yielded 387 raw longitudinal observations; after harmonization and representative-value selection, 173 effect rows from 31 analytic study IDs (29 primary reports) contributed to the trajectory synthesis. Evidence was concentrated in anthracycline-based and anthracycline plus concurrent/sequential HER2 inhibitor settings, whereas data on immune checkpoint inhibitor (ICI) therapy, vascular endothelial growth factor receptor tyrosine kinase inhibitor (VEGF-TKI) therapy, and the biomarkers sST2 and GDF-15 were sparse. In evidence-rich settings, hs-cTnI tended to show early elevations (D0–M3), while more pronounced declines in LVEF and GLS were observed mainly after M3. Because estimates were derived from aggregated study-level data, this ordering is descriptive and hypothesis-generating rather than evidence of within-patient temporal precedence. Under anthracycline plus concurrent/sequential HER2 inhibitor exposure, hs-cTnI elevations and later imaging declines were greater in the available windows; data beyond 12 months were sparse, making the apparent absence of recovery provisional.

**Conclusion:**

This review provides a unified time-window framework for multimarker longitudinal evidence across anticancer treatment settings, identifies critical evidence gaps, and describes an exploratory temporal pattern that may inform hypotheses about stage-specific monitoring in evidence-rich settings. Patient-level longitudinal studies are required before any monitoring schedule can be validated.

**Systematic Review Registration:**

https://www.crd.york.ac.uk/prospero/display_record.php?ID=CRD420261367699, PROSPERO CRD420261367699.

## 1 Introduction

With advances in anticancer therapies, long-term survival rates among patients with cancer have improved substantially (1). However, cancer therapy–related cardiac dysfunction (CTRCD) has emerged as a major factor limiting quality of life and long-term prognosis in this population (2). The clinical manifestations of CTRCD range from asymptomatic signals of myocardial injury to fulminant myocarditis, and even refractory heart failure, and in severe cases, may necessitate interruption of essential anticancer treatment (3). Therefore, early identification of cardiotoxicity and timely implementation of precise interventions remain central challenges in cardio-oncology.

Current monitoring strategies for CTRCD rely mainly on two pillars: cardiac biomarkers, such as troponins and natriuretic peptides, and cardiovascular imaging parameters, such as left ventricular ejection fraction (LVEF) and global longitudinal strain (GLS) (4). Although multiple guidelines and expert consensus statements recommend combining these markers to improve detection, monitoring in clinical practice remains fragmented, with different indicators often interpreted in isolation and their dynamic changes lacking systematic integration (5). Traditional static threshold-based criteria obscure the dynamic nature of CTRCD, which is not a transient event occurring at a single time point, but rather a continuous pathological process that evolves over the course of treatment exposure and follow-up (6).

The cardiotoxicity profiles and temporal scales of onset differ substantially across anticancer treatment classes. Anthracycline-based therapy is more commonly associated with early structural myocardial injury signals (7), whereas HER2 inhibitor–related changes often manifest as fluctuations in cardiac function with potential reversibility (8). In contrast, immune-based therapies may lead to relatively acute inflammatory myocardial events (9). This temporal heterogeneity suggests that monitoring strategies based on a single marker, a single time point, or a uniform follow-up schedule may not adequately reflect the dynamic evolution of cardiotoxicity across different treatment settings (10).

Although the notion that biochemical biomarkers may change earlier than imaging markers has been suggested in clinical experience and single-center studies, this temporal relationship has not yet been systematically synthesized across treatment settings and time windows. Moreover, the specific windows of abnormality onset, duration of change, and cross-regimen trajectory differences for different markers remain insufficiently characterized through systematic review–based quantitative integration.

To address this gap, the present study conducted a systematic search and a two-stage screening process to construct an evidence map across treatment modality, monitoring marker, and time window and performed trajectory reconstruction and temporal integration for longitudinal cohorts that met quantitative inclusion criteria, with the aim of systematically characterizing temporal trajectories of biochemical and imaging markers under different treatment settings, exploring whether recognizable temporal patterns exist among marker changes, and delineating the current distribution of evidence across treatment types and time-window coverage.

## 2 Materials and methods

### 2.1 Study design and evidence mapping

This study was designed as a systematic review combined with evidence mapping and was registered with PROSPERO (CRD420261367699). PubMed and Embase were searched from database inception to 1 October 2025. Searches were organized as a biochemical biomarker × treatment-class matrix. Query structure was tailored to each cell: all searches included treatment and biomarker concepts, with cardiotoxicity/cardiac-dysfunction, longitudinal/follow-up, cancer-population, and human-study terms added as appropriate. LVEF and GLS were not searched as stand-alone marker blocks; their longitudinal values were extracted when reported in articles retrieved through the biochemical-biomarker searches. Available PubMed search strings and the corresponding Embase concept-block translation are provided in the Supplementary Methods; two GDF-15 combinations are documented using the preserved biomarker terms together with the corresponding treatment-specific template used for the other biomarkers.

The review question was framed according to PICOS: Population—patients receiving potentially cardiotoxic anticancer therapy; Intervention/Exposure—anthracyclines, HER2-targeted therapy, immune checkpoint inhibitors, vascular endothelial growth factor (VEGF)/vascular endothelial growth factor receptor (VEGFR) inhibitors, or mixed regimens; Comparator—within-cohort baseline; Outcomes—longitudinal changes in hs-cTnI, hs-cTnT, BNP, NT-proBNP, sST2, GDF-15, LVEF, and GLS across predefined time windows; and Study designs—longitudinal studies with baseline and at least one follow-up measurement, including observational cohorts and eligible control, usual-care, placebo, or no-intervention arms of interventional studies.

According to the distribution and evidence levels of eligible studies, the available evidence was organized and stratified across three dimensions: monitoring marker, time window, and treatment group. Evidence heatmaps were generated using R software (v4.4.1), in which bubble size represented the number of included studies (*k*). To reflect the extent to which longitudinal evidence supported time-course analysis across different treatment regimen–marker combinations, we applied a custom four-level evidence grading system ranging from 0 to 3. This grading system was based primarily on the completeness of longitudinal data along the time axis and the extractability of quantitative values, rather than on conventional hierarchies of study design. Grade 3 indicated studies providing specific numerical values at ≥2 follow-up time points and therefore directly suitable for trajectory reconstruction. Grade 2 indicated longitudinal monitoring with only partial numerical data or incomplete reporting. Grade 1 indicated qualitative trend descriptions without extractable quantitative data. Grade 0 indicated no usable longitudinal monitoring evidence. In the evidence map, color intensity represented the median evidence level within each cell. This custom four-level evidence grade was used to describe data completeness and extractability, to guide inclusion into the quantitative synthesis, and to determine the visual density of the evidence map. It was not used to weight effect sizes, which were weighted by sample size in the trajectory reconstruction, and it is distinct from the formal risk-of-bias assessment described below. Additional details are provided in the legend of Supplementary Table S1.

### 2.2 Cohort definition and data stratification

To characterize the temporal features of cardiotoxicity across different treatment regimens, included cohorts were classified into the following six subgroups:

Anthracyclines: patients receiving chemotherapy regimens containing anthracyclines, such as doxorubicin or epirubicin, without concurrent or sequential HER2-targeted therapy. This group could include patients also receiving other chemotherapeutic agents, such as cyclophosphamide or taxanes, or radiotherapy.

HER2-targeted: patients receiving anti-HER2 therapy alone, such as trastuzumab, pertuzumab, or related monoclonal antibodies or antibody-drug conjugates, without anthracycline exposure.

Anthracyclines + HER2i: patients receiving anthracycline-containing regimens together with HER2-targeted therapy, either concurrently or sequentially.

Immune checkpoint inhibitor (ICI): patients receiving PD-1/PD-L1 or CTLA-4 inhibitors.

VEGF-TKI: patients receiving vascular endothelial growth factor receptor tyrosine kinase inhibitors such as sunitinib or sorafenib.

Mixed/unclear: cohorts involving mixed treatment regimens from which subgroup-specific data could not be independently extracted, or cohorts with insufficiently specified treatment descriptions.

### 2.3 Two-stage screening and data extraction

A two-stage screening process was applied. First, studies were screened and stratified using the evidence mapping worksheet. Numerical extraction and standardization were performed only when studies met the criteria for quantitative synthesis. Studies falling outside the study scope were recorded in the screening worksheet only and were not included in the quantitative synthesis.

#### 2.3.1 Stage 1: Study screening and eligibility criteria for quantitative inclusion

Studies were eligible only if all of the following criteria were met:

1. The same cohort had both baseline and at least one follow-up measurement.
2. Follow-up time could be mapped to specific calendar days and assigned to a prespecified time window.
3. Extractable longitudinal absolute values were reported, including mean ± SD, median [IQR], or clearly tabulated numerical values.

Studies were excluded if they met any of the following criteria:

- Only trend plots were reported without extractable numerical values; only relative changes were reported; or only model-based statistics were provided, such as odds ratios, hazard ratios, or regression coefficients;
- Outcome-driven cohorts were used, defined as studies reporting data only for subgroups with adverse events, such as myocarditis, heart failure, or reduced ejection fraction. In principle, such studies were excluded from the main synthesis and were considered only for sensitivity analysis or qualitative description when necessary;
- Follow-up time was reported only as vague windows or stage-based descriptions and could not be mapped to specific days;
- The study was outside the scope of the present review.

#### 2.3.2 Stage 2: Quantitative extraction, treatment stratification, deduplication, and selection of control arms

Only studies involving the following biomarkers were included: hs-cTnI, hs-cTnT, BNP, NT-proBNP, sST2, and GDF-15. For imaging markers, only studies involving LVEF and GLS were included.

Treatment exposure was classified according to current treatment as Anthra, HER2i, Anthra + HER2i (concurrent/sequential combination), ICI, VEGF-TKI, or mixed/unclear. Previous treatment history was recorded but did not alter subgroup assignment.

For studies with multiple intervention arms, only the control, usual care, placebo, or no-intervention arm was included in the quantitative synthesis.

For duplicate publications from the same clinical cohort, the report with the larger sample size or more complete follow-up was prioritized. When multiple measurements were reported within the same time window in a single study, only one representative value was retained, preferentially the measurement closest to the midpoint of the time window; if this could not be determined, the latest measurement within that window was used. The representative-value rule was tested against three alternatives (earliest, latest, and within-window mean); the results are reported in Supplementary Figure S3 and Supplementary Table S2 (“Selection sensitivity” and “Peak timing sensitivity” sheets). This ensured that each study contributed only one effect size per marker per time window.

Follow-up time was uniformly converted into days using treatment initiation as the time anchor and then mapped to standard time windows. Data anchored to adverse events were recorded separately and were not mixed into the main analysis. Convertible units, such as pg/mL and ng/L, were standardized; values that could not be converted were retained in their original units.

We extracted biomarker subtype, reported units, assay platform or manufacturer, detection limits, reference limits, and imaging vendor/software when these details were available in the primary reports. Biomarker subtypes were retained separately, including hs-cTnI vs. hs-cTnT and BNP vs. NT-proBNP. Every conversion was documented: median [IQR] → mean/SD via Wan et al. (20), cross-checked against Luo et al. (21); mean (95% CI) → SD via SD = √n × (upper − lower)/(2 × 1.96); and unit harmonization (pg/mL ≡ ng/L, with verification that the 3 ng/mL troponin studies never entered a fold change). Skewed biochemical markers were expressed as log2 fold-changes to place proportional increases and decreases on a symmetric scale. Uncertainty introduced by converting reported medians/IQRs to means/SDs was retained as a limitation. A per-record conversion-rule column is provided in Supplementary Table S1 so that every extracted value is traceable to its exact transformation. Assay or platform information that was not explicitly reported was recorded as “Not reported” rather than inferred; an independent assay-platform audit is provided as a dedicated sheet in Supplementary Table S1.

The second-stage screening yielded 46 independent cohorts and 387 raw longitudinal observations. After harmonization and record-level documentation, Supplementary Table S1 contains 372 source-level extraction records. After application of the analytic eligibility and representative-value rules, the effect dataset used for quantitative trajectory summaries comprised 173 effect rows across 31 analytic study IDs from 29 primary reports. The risk-of-bias assessment was performed at the primary-report/study-record level, comprising 29 judgment records covering all 31 analytic study IDs; this denominator should not be interpreted as the number of raw observations, harmonized extraction rows, or analytic effect rows.

### 2.4 Time-course evidence synthesis and statistical analysis

#### 2.4.1 Effect size calculation and direction definition

For biochemical biomarkers, the effect size was calculated as follows:

$$\log2\mathrm{FC}=\mathrm{lo}g_{2}\left(\frac{\mathrm{Follow}\text{-}\mathrm{up}}{\mathrm{Baseline}}\right)$$

A positive value (log2FC > 0) indicated an increase in concentration. For zero values or values below the lower limit of detection (LLOD), the original study-specific handling rule was followed whenever available. If no explicit rule was reported, substitution with the minimum detectable value was applied and recorded in the extraction table. Below-detection handling was further tested in sensitivity analyses using alternative rules, including substitution with the minimum detectable value, fixed floors at LLOD = 2 and LLOD = 5, and exclusion of near-LLOD baselines; the results are reported in Supplementary Figure S4 and Supplementary Table S2 (“LLOD sensitivity” sheet).

For imaging markers:

LVEF:

$$\text{\%}\mathrm{Change}=\frac{\mathrm{Follow}\text{-}\mathrm{up}\text{-}\mathrm{Baseline}}{\mathrm{Baseline}}\;\times 100\text{\%}$$

A negative value indicated a decrease, corresponding to worsening function.

GLS:

Absolute GLS values, |GLS|, were used uniformly for calculation:

$$\text{\%}\mathrm{Chang}e_{\mathrm{GLS}}=\frac{|\mathrm{GL}S_{\mathrm{follow}\text{-}\mathrm{up}}|-|\mathrm{GL}S_{\mathrm{baseline}}|}{|\mathrm{GL}S_{\mathrm{baseline}}|}\times 100\text{\%}$$

A negative value indicated a decrease in |GLS|, corresponding to worsening strain.

#### 2.4.2 Trajectory reconstruction and visualization

Using R (v4.4.1), study-level effect estimates were aggregated within each predefined time window to calculate the central trend:

$${\bar{X}}_{w}=\frac{\sum n_{i}x_{i}}{\sum n_{i}}$$

where $x_{i\;}$ denotes the study-specific effect size and *n*_i_ denotes the corresponding study-specific sample size within that time window. Bubble size was proportional to the total sample size within each time window, with an upper limit of *n* = 200 to optimize visualization. Time windows supported by relatively few studies were marked with hollow circles. Window-specific uncertainty was reported using non-parametric bootstrap 95% confidence intervals based on 2,000 resamples; windows informed by a single study were drawn with hollow markers and no confidence interval band (Supplementary Figure S1; Supplementary Table S2, “CI estimates” and “Central trends” sheets).

Where *k* ≥ 3, between-study heterogeneity was quantified using DerSimonian–Laird random-effects models, with *τ*^2^, *I*^2^, and 95% prediction intervals reported in Supplementary Figure S2 and Supplementary Table S2 (“Heterogeneity” sheet).

We used sample-size-weighted aggregation of study-level effect estimates within prespecified windows as a descriptive summary of central tendency. This choice reflects the structure of the available data: studies report at irregular and non-overlapping follow-up times, in heterogeneous formats, and rarely provide the within-patient covariance across time points that a longitudinal model would require. The method therefore rests on the following assumptions and carries the following limits. (i) It treats each study-window effect as an independent contribution and does not model within-study dependence across repeated windows; (ii) it does not, in its point estimate, incorporate between-study heterogeneity or differential study quality; and (iii) the weighting reflects sample size and not study precision or risk of bias. Accordingly, the reconstructed trajectories support descriptive and hypothesis-generating statements about the shape and timing of marker changes, but they do not support confirmatory pooled effect estimation, formal between-group hypothesis testing, or causal inference. To characterize—rather than assume away—these limitations, we additionally report (a) non-parametric bootstrap 95% confidence intervals around each window estimate (Supplementary Figure S1), (b) DerSimonian–Laird random-effects heterogeneity metrics (*τ*^2^, *I*^2^, prediction intervals) for windows with *k* ≥ 3 (Supplementary Figure S2 and Supplementary Table S2), and (c) sensitivity analyses to the value-selection rule and to below-detection handling (Supplementary Figures S3, S4). Funnel-based publication-bias assessment was not undertaken because the small number of studies per window and the single-arm, within-study fold-change structure would make such analyses unreliable and difficult to interpret.

### 2.5 Risk-of-bias assessment

We performed a formal risk-of-bias assessment using a ROBINS-I–adapted framework tailored to single-arm or non-randomized longitudinal biomarker cohorts. Risk of bias was assessed at the primary-report/study-record level rather than at the extraction-row level; multiple biomarker, time-window, or cohort extraction rows from the same primary report were mapped to a single risk-of-bias judgment where appropriate. The assessment considered six domains: confounding, cohort selection, exposure classification, missing follow-up or survivor bias, outcome measurement or assay, and selective reporting of time points. Each domain and the overall judgment were classified as Low, Some concerns/Moderate, High/Serious, or Unknown. When a primary report did not provide sufficient information to support a defensible judgment, the domain was classified as Unknown rather than inferred. This risk-of-bias assessment is distinct from the custom evidence grading: the custom grading evaluated data completeness and extractability for trajectory reconstruction, whereas the risk-of-bias assessment addressed study-level limitations relevant to internal validity. The judgment table, supporting audit notes, and heatmap are provided in Supplementary Figure S5 and Supplementary Table S3.

## 3 Results

**Figure 1:**
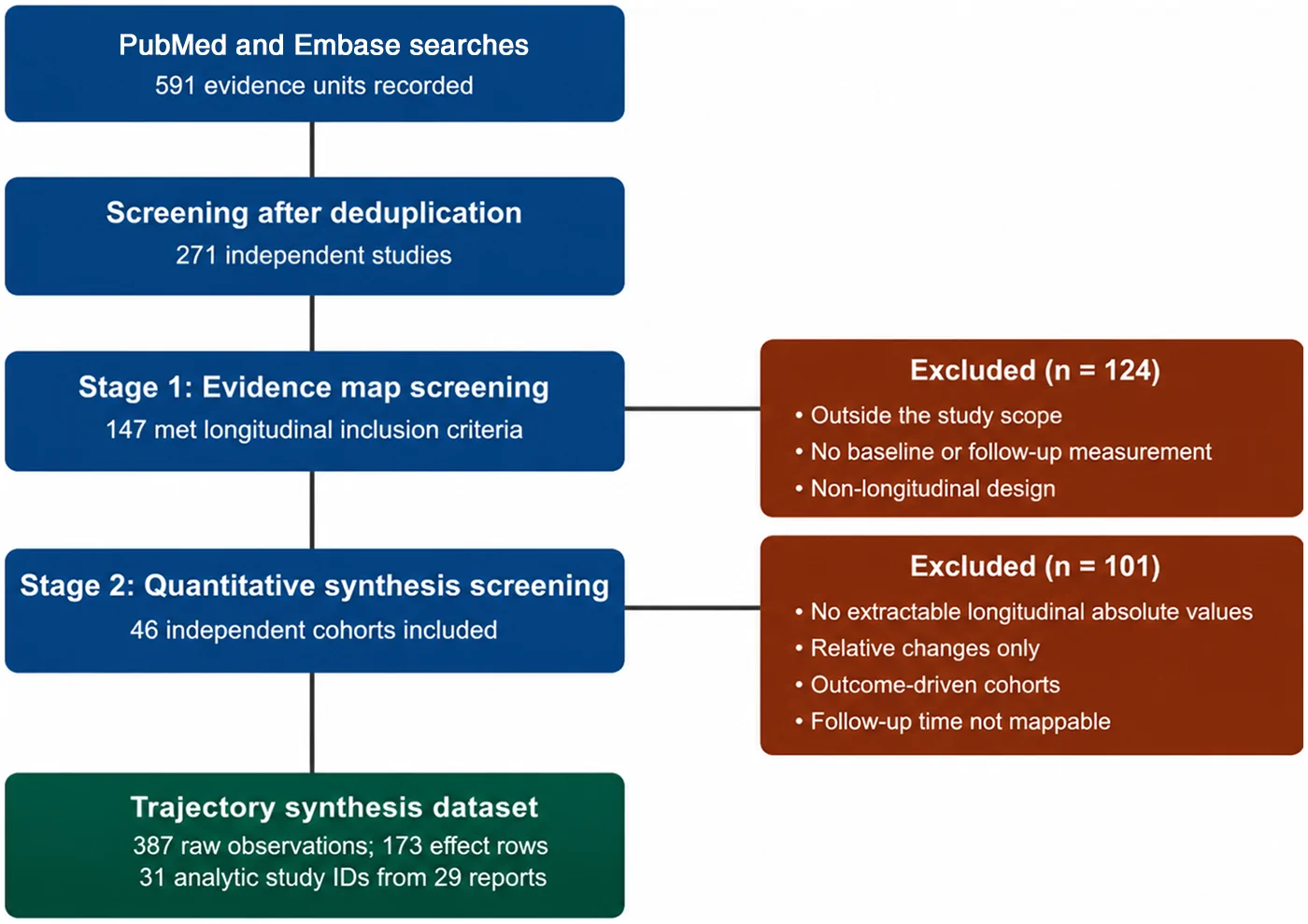
A flow diagram of study screening and quantitative inclusion.

During the item-by-item screening of the evidence-mapping worksheet, a total of 591 evidence units were recorded. After deduplication, 271 independent studies were screened, of which 147 met the longitudinal inclusion criteria for evidence mapping and proceeded to subsequent data extraction. In the second-stage screening, 46 independent cohorts yielded 387 raw longitudinal observations. After harmonization and representative-value selection, 173 effect rows across 31 analytic study IDs from 29 primary reports contributed to the quantitative trajectory summaries. The study screening and quantitative inclusion process is summarized in Figure 1.

### 3.1 Distribution of evidence: polarization and evidence gaps

**Figure 2:**
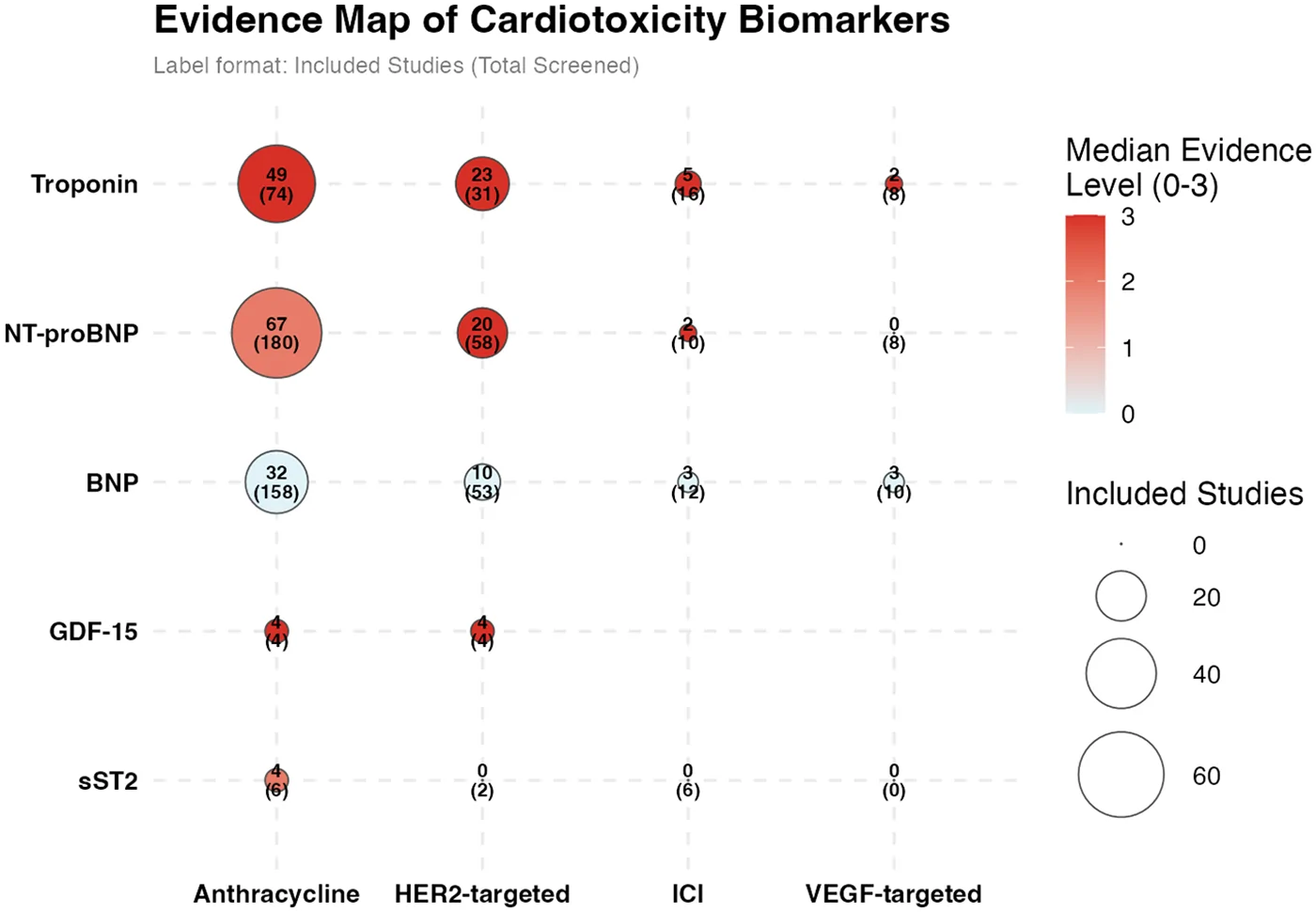
Evidence map of the included studies.

Evidence heatmapping (Figure 2) was used to visualize the distribution of included studies. The results showed a markedly polarized distribution of the current evidence for CTRCD monitoring across treatment regimens and monitoring markers.

Longitudinal evidence was concentrated primarily in the anthracycline-only group, in which studies on NT-proBNP were the most abundant (*k* = 67/180), followed by troponins (*k* = 49/74). These areas also tended to have higher overall evidence levels, although substantial variation remained across studies in design and follow-up strategy. The HER2-targeted therapy group ranked second, but data points were relatively sparse for certain markers or specific time windows. In addition to the limited number of studies, some evidence gaps also arose from inconsistencies in follow-up design and reporting practices, with some studies not assessing or not reporting early time windows.

By contrast, longitudinal monitoring evidence was relatively sparse in the ICI and VEGF-TKI groups and was largely confined to short-term follow-up. Studies reporting time-course measurements of emerging biomarkers such as sST2 and GDF-15 were also limited across treatment groups. These evidence gaps may partly reflect the lack of uniformity in reporting formats and follow-up definitions in the existing literature, resulting in insufficient longitudinal absolute values and comparable time points for quantitative synthesis, such as the absence of extractable absolute values or follow-up times that could not be mapped to specific days. However, a higher density of evidence did not necessarily indicate greater consistency of findings. Even in some relatively evidence-rich areas, such as biochemical markers related to anthracycline therapy, appreciable between-study heterogeneity remained.

### 3.2 Temporal trajectories of biochemical biomarkers

#### 3.2.1 Markers of myocardial injury (hs-cTnI and hs-cTnT)

**Figure 3:**
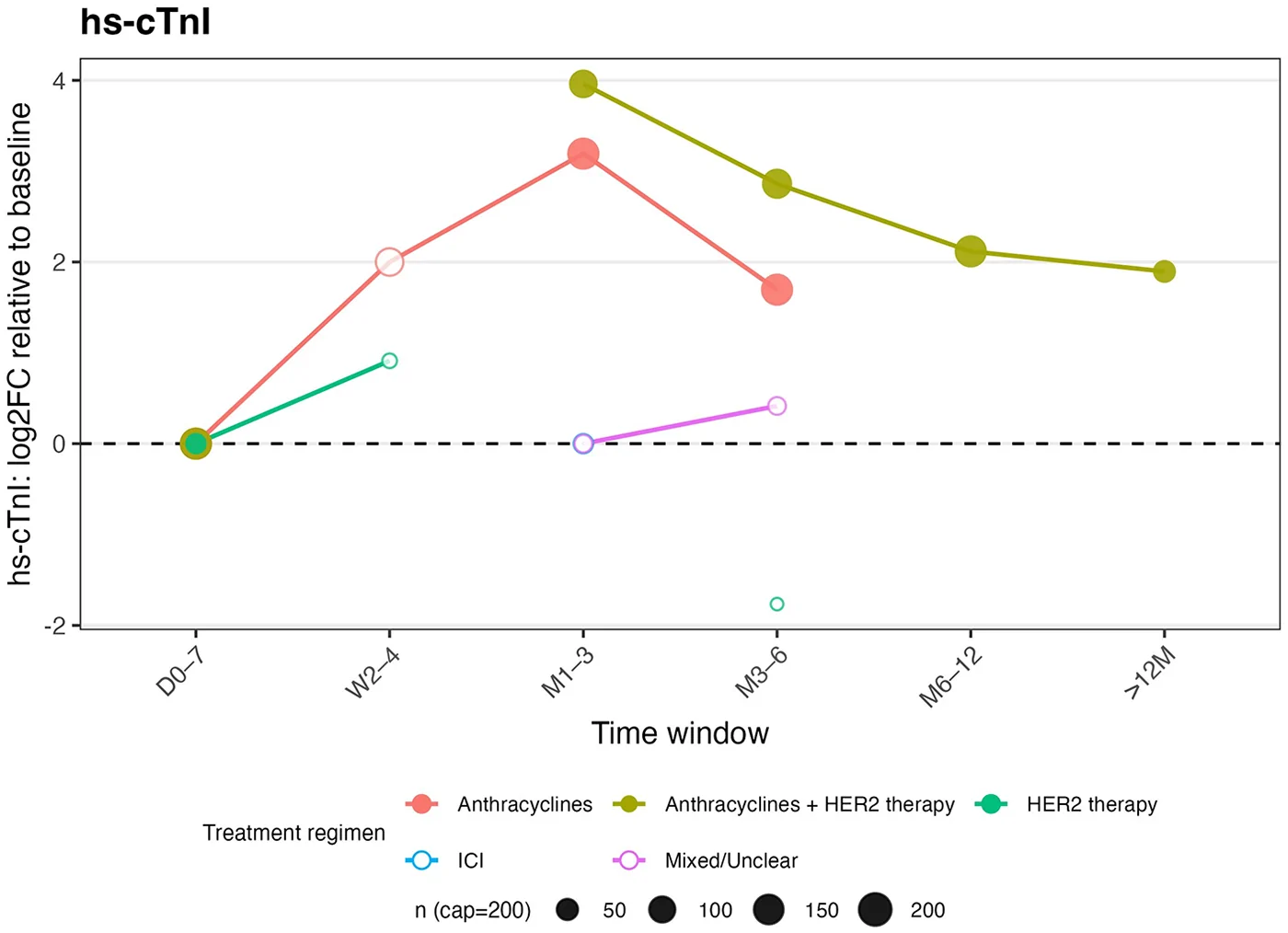
Temporal trajectory of hs-cTnI across different treatment regimens. Hollow markers denote windows supported by a single study (*k* = 1). Total *n* and *k* per window are provided in the evidence-grade sheet of Supplementary Table S1 and in the “CI estimates” sheet of Supplementary Table S2.

**Figure 4:**
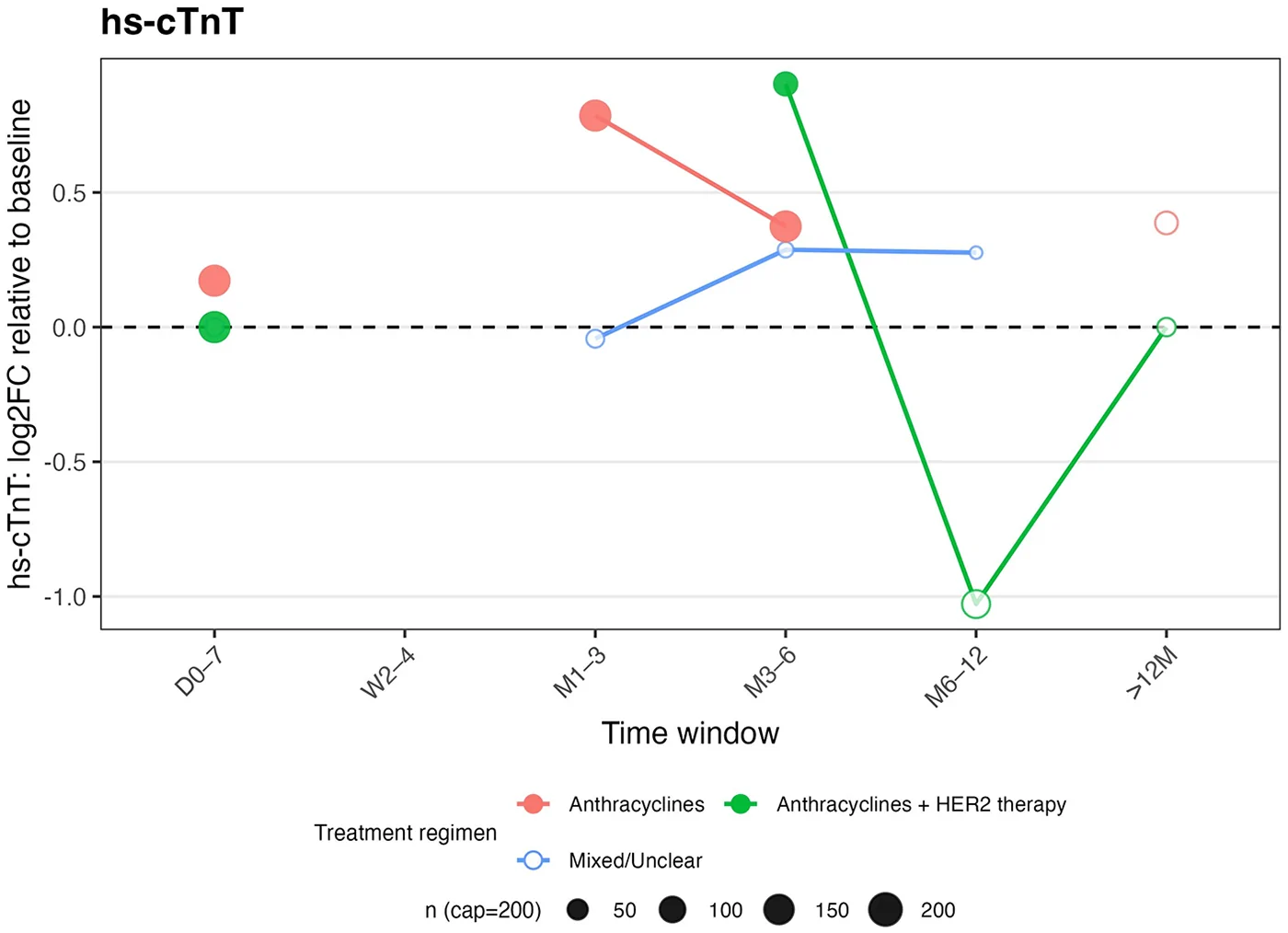
Temporal trajectory of hs-cTnT across different treatment regimens. Hollow markers denote windows supported by a single study (*k* = 1). Total *n* and *k* per window are provided in the evidence-grade sheet of Supplementary Table S1 and in the “CI estimates” sheet of Supplementary Table S2.

High-sensitivity cardiac troponin I (hs-cTnI) and T (hs-cTnT) showed distinct temporal patterns across different treatment settings (Figures 3, 4).

In the anthracycline-only group, hs-cTnI exhibited a characteristic single-peak increase during the M1–3 time window, followed by a decline, indicating that anthracycline-related myocardial injury signals were more prominent in the early post-treatment period. In the anthracyclines plus concurrent/sequential HER2 inhibitor group, the increase in hs-cTnI was greater in magnitude and declined more slowly, remaining above baseline even at the >12M time window, suggesting that hs-cTnI elevation persisted across the available windows under combined treatment exposure.

The less consistent hs-cTnT trajectory (Figure 4) should be interpreted cautiously and likely reflects a combination of factors rather than a genuine biological divergence from hs-cTnI. First, data density: hs-cTnT contributed fewer studies and smaller per-window sample sizes compared with hs-cTnI; hence, several hs-cTnT windows rest on one or two cohorts (hollow markers, Figure 4; Supplementary Table S1). Second, assay heterogeneity: hs-cTnI and hs-cTnT are measured on different, non-interchangeable platforms with different baseline distributions and limits of detection (documented in the assay-platform audit sheet of Supplementary Table S1); hence, cross-study pooling of hs-cTnT is more sensitive to assay differences. Third, baseline-proximity/LLOD effects: several hs-cTnT baselines were low but not as close to the detection limit as some hs-cTnI baselines, changing the leverage of fold-change computation (see LLOD sensitivity analysis, Supplementary Figure S4). We therefore do not interpret the hs-cTnT–hs-cTnI difference as evidence of distinct biology; it is most parsimoniously explained by data availability and measurement heterogeneity.

#### 3.2.2 Marker of cardiac load (NT-proBNP)

Although both BNP and NT-proBNP were eligible, the trajectory synthesis is presented for NT-proBNP only. This reflects data availability and comparability rather than a biological preference: extractable longitudinal BNP series meeting the inclusion criteria were too few to reconstruct a stable trajectory, and BNP and NT-proBNP are not numerically interchangeable (different molecular targets, assays, and reference ranges), and therefore, they could not be pooled. BNP is therefore retained in the evidence map and Supplementary Table S1 (including the assay-platform audit) for completeness but is not synthesized as a trajectory.

**Figure 5:**
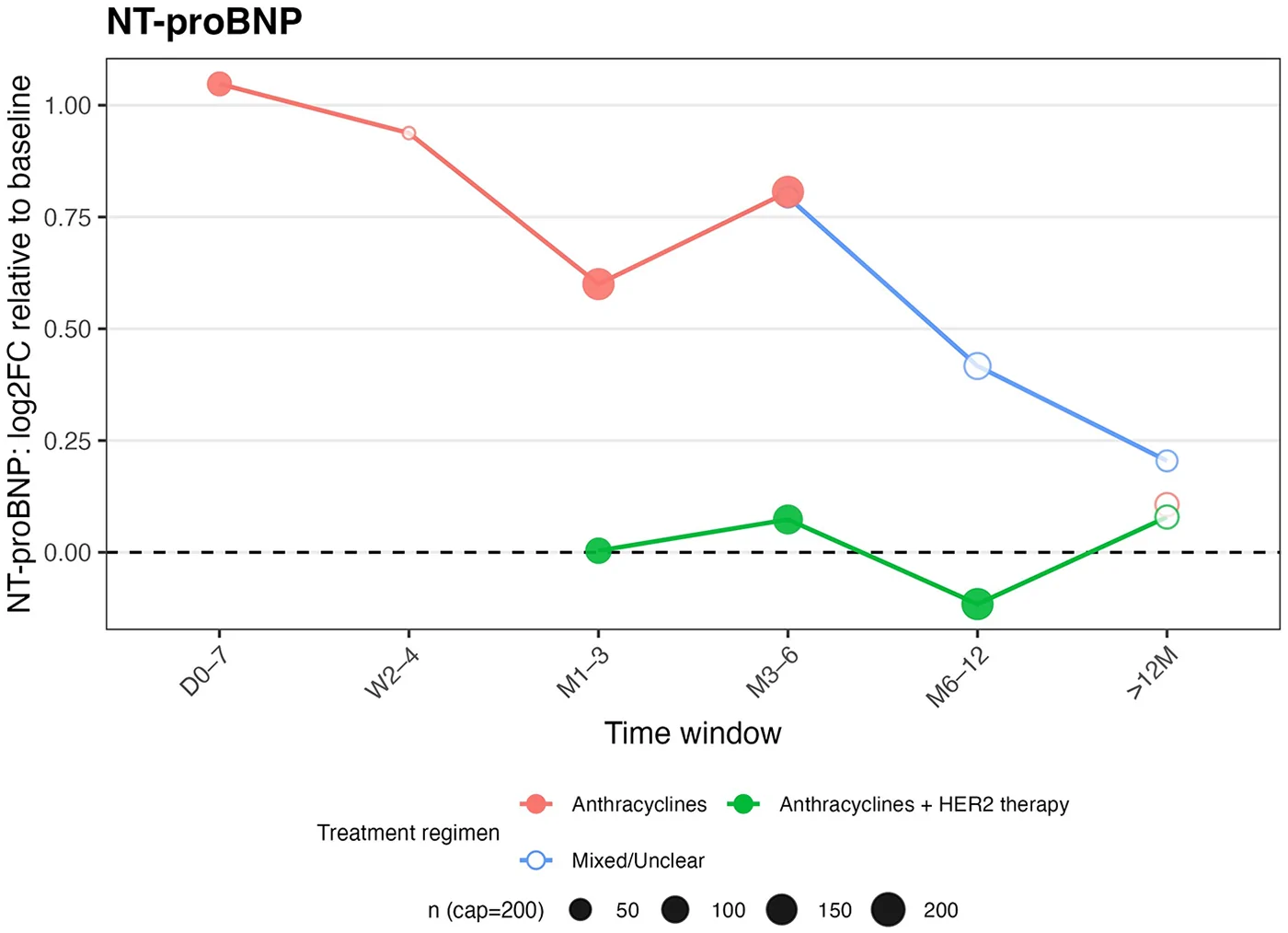
Temporal trajectory of NT-proBNP across different treatment regimens. Hollow markers denote windows supported by a single study (*k* = 1). Total *n* and *k* per window are provided in the evidence-grade sheet of Supplementary Table S1 and in the “CI estimates” sheet of Supplementary Table S2.

The temporal trajectory of NT-proBNP showed marked heterogeneity across different treatment settings (Figure 5).

The anthracycline-only group showed a relatively consistent early stress pattern, with NT-proBNP reaching a peak increase during the W2–4 time window and then gradually declining, although overall levels generally remained above baseline, suggesting that increased volume load or ventricular wall stress may emerge early during treatment and persist over longer follow-up.

In the anthracyclines plus concurrent/sequential HER2 inhibitor group, NT-proBNP remained overall close to baseline across the available time windows and showed non-monotonic fluctuations, without a sustained and consistent upward trend. Data points in the early time windows were sparse in this subgroup, limiting the stability of inferences regarding its early-phase pattern. The Mixed/Unclear group showed a midterm increase followed by a gradual decline.

### 3.3 Temporal trajectories of imaging-based functional markers

#### 3.3.1 Left ventricular ejection fraction

**Figure 6:**
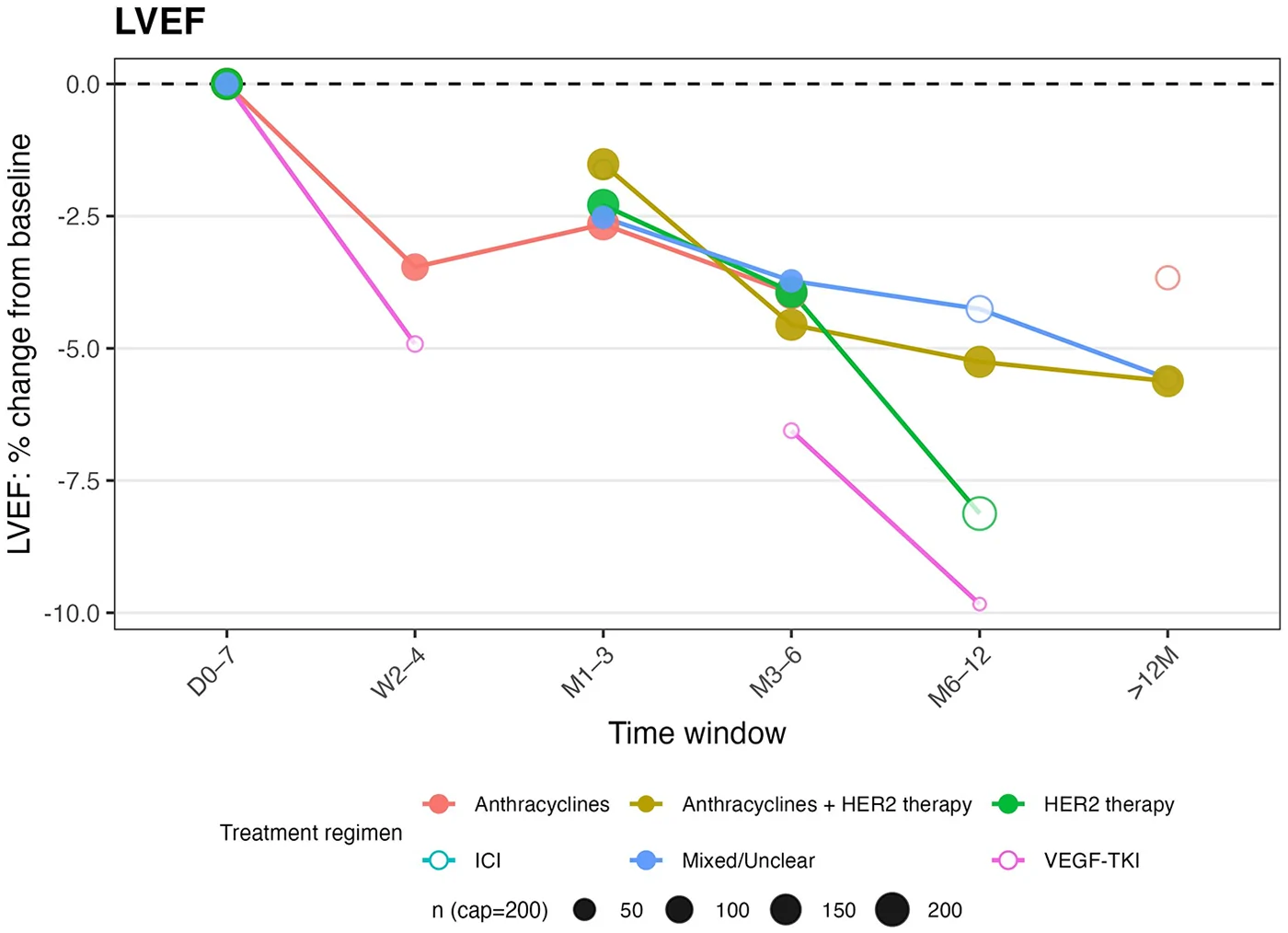
Temporal trajectory of LVEF across different treatment regimens. Hollow markers denote windows supported by a single study (*k* = 1). Total *n* and *k* per window are provided in the evidence-grade sheet of Supplementary Table S1 and in the “CI estimates” sheet of Supplementary Table S2.

The temporal pattern of LVEF clearly delineated the window of systolic functional impairment across different treatment regimens (Figure 6). In the anthracycline-only group, LVEF showed a moderate decline followed by relative stabilization over time.

By contrast, in the anthracycline plus concurrent/sequential HER2 inhibitor group, LVEF declined progressively from M1–3 and further after M3–6. No clear recovery was apparent in the sparse data beyond 12 months, and this finding should therefore be interpreted cautiously.

#### 3.3.2 Global longitudinal strain

GLS showed declines from the M3–6 window onward in the available data, with larger changes than LVEF in some windows; however, the comparative sensitivity of the two imaging markers cannot be established from these aggregated study-level data.

In the HER2-targeted monotherapy group, available time windows were too limited to support a stable trajectory. Imaging data were also sparse in the ICI and VEGF-TKI groups.

### 3.4 Temporal integration: an exploratory temporal pattern between myocardial injury signals and functional remodeling

Taking anthracycline therapy and anthracycline plus concurrent/sequential HER2 inhibitor exposure as evidence-rich settings, biochemical injury signals tended to appear earlier than the more pronounced functional changes observed in later windows. This exploratory ordering was reconstructed from aggregated study-level summaries and does not establish within-patient temporal precedence. This pattern could be summarized into two stages. The anthracycline-plus-HER2i group pools concurrent and sequential HER2i schedules anchored to treatment initiation; this limitation is discussed further in Section 4.3.

Early warning phase (D0–M3): This phase was dominated by fluctuations in biochemical markers. The characteristic elevation of hs-cTnI was concentrated mainly within D0–M3, showing a single-peak pattern followed by decline in the anthracycline-only group, whereas in the combined treatment group, the peak was higher and the decline was slower. In the anthracycline-only group, NT-proBNP also frequently deviated from baseline during the W2–4 time window. Although changes in LVEF and GLS were already detectable at this stage, their magnitude was generally smaller than the cumulative changes observed later.

Functional remodeling phase (>M3): Imaging markers gradually became the dominant feature. More pronounced declines in LVEF and GLS occurred mainly during the M3–6 and later time windows. More pronounced declines in LVEF and GLS were observed in the M3–6 and later windows in the anthracycline plus concurrent/sequential HER2 inhibitor group. Data beyond 12 months were sparse, so the apparent absence of recovery remains provisional.

It should be noted that not all markers followed a consistent temporal pattern. The overall trajectory of hs-cTnT showed substantial fluctuation, which may have been influenced by between-study heterogeneity and limited sample size. In the concurrent/sequential treatment group, NT-proBNP did not show a sustained and consistent increase across the available time windows, and its interpretation should therefore be considered in light of the methodological issues discussed in Section 4.3. Moreover, for ICI, VEGF-TKI, and emerging biomarkers, the currently available longitudinal data remain too sparse to support stable inference.

Formal quantification (Supplementary Figure S2 and Supplementary Table S2) showed high between-study heterogeneity in most windows (*I*^2^ > 75% in 18 of 29 windows with *k* ≥ 3), which further argues for a descriptive rather than confirmatory interpretation. The consistency of the qualitative pattern was, however, supported by sensitivity analyses to the representative-value selection rule and to below-detection handling (Supplementary Figures S3, S4).

### 3.5 Risk-of-bias assessment

Risk-of-bias assessment was completed at the primary-report/study-record level. The final assessment comprised 29 risk-of-bias judgement records, covering all 31 analytic study IDs contributing to the effect dataset (two primary reports each contributed two analytic cohorts). At the judgment-record level, all 29 records were judged as having Some concerns/Moderate risk of bias overall; no judgment record was rated Low, High/Serious, or Unknown overall. Four individual domain-level judgments remained Unknown because the corresponding primary reports did not provide sufficient information for a defensible assessment. The main sources of concern were incomplete reporting of confounding variables, incomplete exposure timing or treatment-sequence information in some cohorts, missing follow-up and potential survivor bias (particularly in longer follow-up windows), assay or platform heterogeneity and incomplete assay reporting, and selective or sparse reporting of time points. These findings further support interpreting the reconstructed biomarker and imaging trajectories as descriptive and hypothesis-generating, rather than as confirmatory pooled evidence.

## 4 Discussion

### 4.1 Capturing early signals: temporal concordance between biochemical abnormalities and mechanistic insights

The time-course analysis in this study showed that hs-cTnI in the anthracycline-treated group exhibited a characteristic peak from D0–7 to M1–3 (Figure 3). This early window is consistent with the known time scale of anthracycline-related myocardial injury mechanisms. After entering the body, anthracyclines intercalate into DNA and poison topoisomerase IIβ (Topo IIβ), thereby inducing double-strand breaks and transcriptional dysregulation (11). At the same time, quinone/semiquinone redox cycling generates large amounts of reactive oxygen species, triggering lipid peroxidation (12). These molecular events may be more active during the early phase after drug administration, leading to increased cardiomyocyte membrane permeability and troponin release (13). Accordingly, the early elevation of hs-cTnI may be viewed as a timely molecular-level reflection of myocardial injury, whereas changes in functional markers such as LVEF and GLS may emerge more gradually after damage has accumulated. This observed temporal correspondence is therefore compatible with the pathological progression from microscopic injury to macroscopic dysfunction. However, because the present study was based on group-level time-course synthesis, this correspondence should be regarded only as a pathophysiologically plausible inference rather than direct evidence of a causal chain linking molecular events to biomarker changes.

Notably, in the combined treatment group, hs-cTnI not only reached a higher peak but also remained elevated at M3–6 and even >12M. This suggests that in sequential treatment settings, beyond the initial insult caused by anthracyclines, HER2 inhibitors may further impair cardiomyocyte self-repair and survival signaling by blocking the Neuregulin-1/ErbB pathway, thereby contributing to the persistence of subclinical myocardial stress (14). Within the framework of this study, hs-cTnI may therefore serve not only as a signal of acute injury, but also, through its longer-term trajectory, as a potential reflection of disrupted repair-related pathways.

Compared with hs-cTnI, the temporal behavior of NT-proBNP in this study appeared to be more context-dependent. In the anthracycline-only group, NT-proBNP generally showed an early increase followed by a decline, although levels often remained above baseline, suggesting that it may reflect early increases in ventricular wall tension or myocardial stress rather than direct cardiomyocyte injury *per se*. In other words, the biological information conveyed by NT-proBNP is not identical to that reflected by hs-cTnI: the former is more closely related to load and stress responses, whereas the latter more directly reflects myocardial injury. Therefore, in anthracycline-related settings, early abnormalities in these two markers may be understood as complementary signals of distinct pathological processes.

### 4.2 Additive risk under combined therapy: delayed deterioration of functional markers

**Figure 7:**
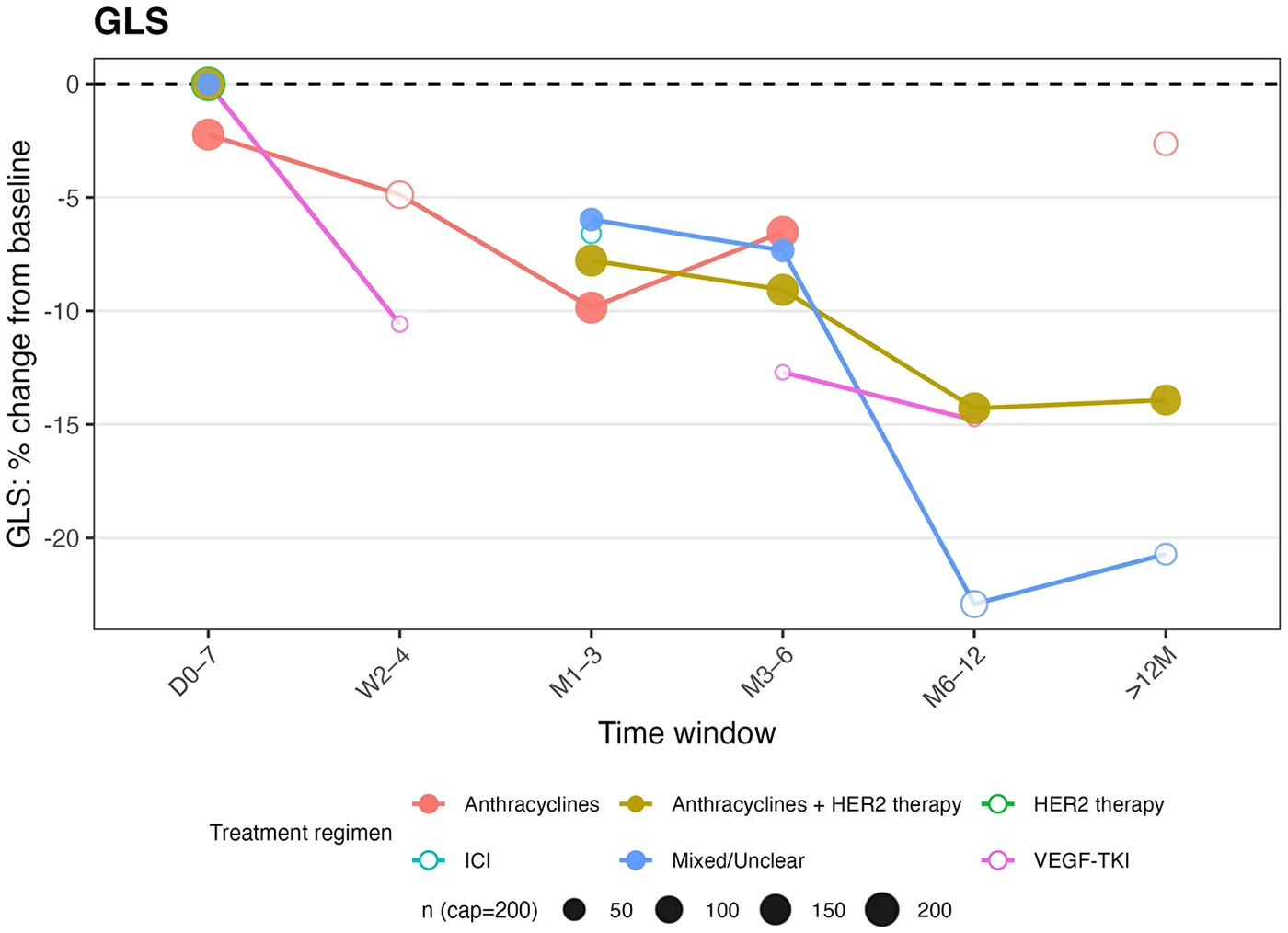
Temporal trajectory of GLS across different treatment regimens. Hollow markers denote windows supported by a single study (*k* = 1). Total *n* and *k* per window are provided in the evidence-grade sheet of Supplementary Table S1 and in the “CI estimates” sheet of Supplementary Table S2.

The imaging trajectories (Figures 6, 7) were consistent with an exploratory temporal pattern in which changes in myocardial injury signals were observed earlier than the more pronounced functional remodeling; because these trajectories were reconstructed from aggregated study-level data, this ordering is descriptive and does not establish within-patient temporal precedence. Although biochemical injury signals generally peaked at M1-3, more pronounced declines in LVEF and GLS were observed mainly after M3-6 in the available windows, particularly in the combined-treatment group. This observed temporal separation is compatible with, but does not establish, a stepwise pathological process linking microscopic cellular injury to overt impairment of pump function.

The greater long-term decline in LVEF observed in the combined treatment group, with no clear recovery apparent in the available follow-up windows, suggests a possible pattern of cumulative functional impairment under a multiple-hit framework. HER2 signaling not only maintains cardiomyocyte metabolic homeostasis but also participates in the response to hemodynamic stress (15). Once anthracyclines have caused the initial structural injury, subsequent HER2 inhibition may further compromise the myocardium's ability to initiate compensatory hypertrophy or repair programs, ultimately leading to sustained depletion of cardiac functional reserve (16).

GLS showed earlier or more pronounced abnormalities in some available windows. However, because the trajectories were reconstructed from aggregated study-level data rather than direct within-patient comparisons, the comparative sensitivity of GLS and LVEF cannot be established here. GLS may nevertheless provide complementary information on early-to-intermediate functional remodeling under combined-treatment exposure.

### 4.3 Boundaries of the evidence and study limitations

This study reconstructed temporal trajectories from aggregated data and therefore has several limitations. First, the finding that NT-proBNP in the concurrent/sequential treatment group remained overall close to baseline and showed non-monotonic fluctuations across time windows (Figure 5) should not be interpreted as indicating a lower risk of cardiac load-related stress. Rather, this pattern more likely reflects heterogeneity in study design and reporting, as well as potential selection bias. Examples include differences in enrollment criteria, with some HER2-targeted therapy studies strictly excluding patients with elevated baseline BNP or prior heart failure, thereby lowering the overall risk profile of the follow-up cohort (17); follow-up attrition and survivor bias, whereby patients who developed overt cardiac events or marked biomarker elevations may have discontinued treatment, been referred elsewhere, or withdrawn from follow-up, leaving behind an apparently healthier retained sample (18); and missing early time windows together with extractability limitations, because some studies in concurrent/sequential treatment settings did not systematically measure natriuretic peptides during the early treatment phase or did not report them as extractable longitudinal absolute values, thereby preventing their inclusion in the main quantitative synthesis and limiting stable inference regarding early peak patterns (19).

In addition, this study applied strict screening gates and bias control to the quantitative data. A substantial proportion of studies involving ICIs, VEGF-TKIs, or emerging biomarkers reported only dichotomous threshold-based outcomes, risk model estimates, or non-extractable trend plots or provided longitudinal data only in subgroups that developed cardiotoxic outcomes. To avoid directionally biased trajectories and overestimation of toxicity caused by outcome-based selection, these studies were excluded from the main synthesis. Although this strategy reduced the density of data available for time-course reconstruction, particularly limiting stable inference for ICIs, VEGF-TKIs, and emerging biomarkers across different time windows, it also improved the comparability and auditability of the included data, making the resulting trends more reflective of the overall evolution in non–outcome-selected cohorts.

All measurements were anchored to treatment initiation. Consequently, patients falling in the same time window may have been at materially different treatment stages—a limitation that is especially acute in the anthracycline-plus-HER2i group, where concurrent and sequential regimens differ in both the biological timing of exposure and the cumulative dose profile at any given calendar time. Pooling concurrent and sequential schedules under a single initiation anchor may therefore blur genuinely distinct trajectories and may affect not only the absolute magnitude of effects but potentially their apparent timing. We were unable to disaggregate concurrent vs. sequential schedules because most studies did not report exposure timing in an extractable form. Conclusions for the combined-therapy group should be tempered accordingly, and schedule-resolved longitudinal reporting is a priority for future studies.

The risk-of-bias assessment and the custom evidence grading address different aspects of the evidence base. The custom evidence grading described data completeness and extractability for trajectory reconstruction, whereas the ROBINS-I–adapted risk-of-bias assessment evaluated study-level limitations relevant to internal validity. The risk-of-bias findings support a cautious, descriptive, and hypothesis-generating interpretation of the reconstructed trajectories rather than a confirmatory pooled interpretation. Assay and platform heterogeneity also limited direct comparability across studies: although biomarker subtypes (hs-cTnI, hs-cTnT, BNP, NT-proBNP) were retained separately, assay manufacturer, analytical sensitivity, lower detection limits, reference limits, and echocardiographic or strain-analysis software were incompletely reported in several primary studies. Between-study differences in biomarker trajectories may therefore partly reflect differences in assay/platform characteristics and reporting practices, in addition to differences in patient populations, cancer therapies, and follow-up schedules.

Given these limitations, the exploratory dynamic monitoring pattern described in this study is most applicable to the evidence-rich settings of anthracycline therapy and anthracyclines plus concurrent/sequential HER2 inhibitors. Conclusions regarding ICIs, VEGF-TKIs, and some emerging biomarkers should still be interpreted with caution.

### 4.4 Conclusions and clinical implications

In summary, CTRCD should not be regarded as a clinical event occurring at a single time point, but rather as a pathophysiological continuum spanning the entire course of anticancer treatment and characterized by distinct temporal windows. Through a systematic review and time-course evidence synthesis, this study provides a unified time-window framework for integrating longitudinal multimarker trajectories across treatment regimens. The main contributions of this study can be summarized in three aspects.

First, this study systematically characterizes a temporal pattern across the treatment course. In the evidence-rich settings of anthracycline therapy and concurrent/sequential HER2 inhibitor exposure, hs-cTnI showed early elevations, whereas the more pronounced deterioration of imaging-based functional markers (LVEF, GLS) was observed in later windows. Reconstructed from aggregated study-level rather than patient-level data, this ordering is a hypothesis-generating observation and does not establish within-patient temporal precedence. It nonetheless organizes previously scattered observations into a time-anchored body of evidence that may inform hypotheses about monitoring windows for different markers.

Second, this study generates a hypothesis for stage-specific dynamic monitoring in evidence-rich settings. Within the two evidence-rich settings only—anthracycline monotherapy and anthracycline plus concurrent/sequential HER2 inhibition—the exploratory temporal pattern is compatible with, and generates the hypothesis for, a stage-specific monitoring approach: (1) during the early phase (D0–M3), biochemical markers such as hs-cTnI may warrant emphasis; and (2) during the mid- to long-term phase (>M3), imaging markers such as GLS and LVEF may warrant emphasis. This is a hypothesis to be tested prospectively and not a validated monitoring schedule, and it should not be extrapolated to ICI, VEGF-TKI, or the emerging biomarkers (sST2, GDF-15), for which the present data are too sparse to support any monitoring recommendation.

Third, it visualizes critical evidence gaps. Longitudinal monitoring evidence for ICI therapy, VEGF-TKI therapy, and emerging biomarkers such as sST2 and GDF-15 remains severely limited and fragmented, and the monitoring windows established for anthracycline-related cardiotoxicity should not be directly extrapolated to these settings. The evidence map generated in this study helps identify priorities for future prospective follow-up studies, particularly with respect to standardized reporting at prespecified time points and the development of longitudinal multimarker cohorts.

## Data Availability

The original contributions presented in the study are included in the article/Supplementary Material, and further inquiries can be directed to the corresponding author.
