# Supplementary material for: Time-dynamic perspectives on cancer therapy–related cardiotoxicity: a systematic review and time-course evidence synthesis for multibiomarker monitoring
Source: Front Cardiovasc Med. 2026 Aug 19;13:1886989. doi: 10.3389/fcvm.2026.1886989 (PMC13535290; doi:10.3389/fcvm.2026.1886989)
Supplement: Supplementary file 1 [file Table1.docx]

**Supplementary Methods — Search Strategy**

*Manuscript ID 1886989 — Frontiers in Cardiovascular Medicine*

The review was registered with PROSPERO (CRD420261367699; registered 13 April 2026). PubMed and Embase were searched from database inception to October 1, 2025. The search date, rather than a publication-date restriction, defined the upper cutoff. Searches were organized as a biochemical biomarker × treatment-class matrix. Query structure was tailored to each cell: all searches included treatment and biomarker concepts, while cardiotoxicity/cardiac-dysfunction, longitudinal/follow-up, cancer-population, and human-study terms were added as appropriate. LVEF and GLS were not searched as standalone marker blocks; their longitudinal values were extracted when reported in articles retrieved through the biochemical-biomarker searches. Sixteen of the 18 biomarker × treatment-class PubMed query strings preserved in the source workbook are reproduced verbatim below. For the two GDF-15 combinations, the preserved biomarker block and the common query template are reported. The Embase searches used the same treatment and biomarker concepts with the corresponding Emtree/Embase syntax (e.g., '/exp', ':ab,ti'), with additional outcome, longitudinal, population, and human-study terms applied as appropriate.

**hs-cTn (high-sensitivity troponin)**

**hs-cTn (high-sensitivity troponin) × Anthracycline (PubMed)**

( "high-sensitivity troponin"[tiab] OR "high sensitivity troponin"[tiab] OR "high-sensitivity cardiac troponin"[tiab] OR "high sensitivity cardiac troponin"[tiab] OR "high-sensitivity cTn"[tiab] OR "high sensitivity cTn"[tiab] OR hs-cTn[tiab] OR hs-cTnI[tiab] OR hs-cTnT[tiab] OR "high-sensitivity troponin I"[tiab] OR "high sensitivity troponin I"[tiab] OR "high-sensitivity troponin T"[tiab] OR "high sensitivity troponin T"[tiab] OR hsTnI[tiab] OR hsTnT[tiab] ) AND ( anthracycline*[tiab] OR doxorubicin[tiab] OR epirubicin[tiab] OR daunorubicin[tiab] OR idarubicin[tiab] ) AND ( cancer[tiab] OR malignan*[tiab] OR tumor*[tiab] OR tumour*[tiab] OR lymphoma[tiab] OR leukemia[tiab] OR leukaemia[tiab] ) AND (human[mh] OR humans[tiab])

**hs-cTn (high-sensitivity troponin) × HER2 inhibitor (PubMed)**

( "high-sensitivity troponin"[tiab] OR "high sensitivity troponin"[tiab] OR "high-sensitivity cardiac troponin"[tiab] OR "high sensitivity cardiac troponin"[tiab] OR "high-sensitivity cTn"[tiab] OR "high sensitivity cTn"[tiab] OR hs-cTn[tiab] OR hs-cTnI[tiab] OR hs-cTnT[tiab] OR "high-sensitivity troponin I"[tiab] OR "high sensitivity troponin I"[tiab] OR "high-sensitivity troponin T"[tiab] OR "high sensitivity troponin T"[tiab] ) AND ( trastuzumab[tiab] OR pertuzumab[tiab] OR "HER2-positive"[tiab] OR "HER2 positive"[tiab] OR HER2[tiab] OR ERBB2[tiab] OR "anti-HER2"[tiab] OR "HER2-targeted"[tiab] ) AND ( breast[tiab] OR "breast cancer"[tiab] OR "breast neoplasm*"[tiab] OR malignan*[tiab] OR tumor*[tiab] OR tumour*[tiab] OR lymphoma[tiab] OR leukemia[tiab] OR leukaemia[tiab] ) AND ( humans[tiab] OR human[mh] )

**hs-cTn (high-sensitivity troponin) × Immune checkpoint inhibitor (PubMed)**

( "Immune Checkpoint Inhibitors"[Mesh] OR "immune checkpoint inhibitor*"[tiab] OR "immune-checkpoint inhibitor*"[tiab] OR ICI[tiab] OR ICIs[tiab] OR "PD-1 inhibitor*"[tiab] OR "PD-L1 inhibitor*"[tiab] OR "CTLA-4 inhibitor*"[tiab] OR nivolumab[tiab] OR pembrolizumab[tiab] OR cemiplimab[tiab] OR atezolizumab[tiab] OR durvalumab[tiab] OR avelumab[tiab] OR ipilimumab[tiab] OR tremelimumab[tiab] ) AND ( "high-sensitivity troponin"[tiab] OR "high sensitivity troponin"[tiab] OR "high-sensitivity cardiac troponin"[tiab] OR "high-sensitivity cardiac troponin I"[tiab] OR "high-sensitivity cardiac troponin T"[tiab] OR "hs-cTnI"[tiab] OR "hsTnI"[tiab] OR "hs-TnI"[tiab] OR "hs-cTnT"[tiab] OR "hsTnT"[tiab] OR "hs-TnT"[tiab] ) AND ( myocarditis[Mesh] OR myocarditis[tiab] OR cardiotoxic*[tiab] OR "cardiac toxicity"[tiab] OR "cardiac injury"[tiab] OR "cardiac adverse event*"[tiab] ) AND ( "Follow-Up Studies"[Mesh] OR "Longitudinal Studies"[Mesh] OR "Time Factors"[Mesh] OR longitudinal[tiab] OR "follow-up"[tiab] OR "follow up"[tiab] OR "serial"[tiab] OR "sequential"[tiab] OR "baseline and"[tiab] OR "baseline, and"[tiab] OR "dynamic change*"[tiab] OR "time course"[tiab] OR "over time"[tiab] OR "during treatment"[tiab] )

**hs-cTn (high-sensitivity troponin) × VEGF/VEGFR inhibitor (PubMed)**

( "Angiogenesis Inhibitors"[Mesh] OR "Vascular Endothelial Growth Factors"[Mesh] OR "VEGF inhibitor*"[tiab] OR "anti-VEGF"[tiab] OR "angiogenesis inhibitor*"[tiab] OR "vascular endothelial growth factor inhibitor*"[tiab] OR "anti-angiogenic"[tiab] OR bevacizumab[tiab] OR ramucirumab[tiab] OR aflibercept[tiab] OR ziv-aflibercept[tiab] OR sunitinib[tiab] OR sorafenib[tiab] OR pazopanib[tiab] OR axitinib[tiab] OR lenvatinib[tiab] OR cabozantinib[tiab] OR regorafenib[tiab] OR vandetanib[tiab] ) AND ( "high-sensitivity troponin"[tiab] OR "high sensitivity troponin"[tiab] OR "high-sensitivity cardiac troponin"[tiab] OR "high-sensitivity cardiac troponin I"[tiab] OR "high-sensitivity cardiac troponin T"[tiab] OR "hs-cTnI"[tiab] OR "hsTnI"[tiab] OR "hs-TnI"[tiab] OR "hs-cTnT"[tiab] OR "hsTnT"[tiab] OR "hs-TnT"[tiab] ) AND ( "Heart Failure"[Mesh] OR "Ventricular Dysfunction, Left"[Mesh] OR "Cardiotoxicity"[Mesh] OR "heart failure"[tiab] OR "cardiac failure"[tiab] OR "ventricular dysfunction"[tiab] OR LVEF[tiab] OR cardiotoxic*[tiab] OR "cardiac toxicity"[tiab] OR "cardiac injury"[tiab] OR "cardiac adverse event*"[tiab] ) AND ( "Follow-Up Studies"[Mesh] OR "Longitudinal Studies"[Mesh] OR "Time Factors"[Mesh] OR longitudinal[tiab] OR "follow-up"[tiab] OR "follow up"[tiab] OR "serial"[tiab] OR "sequential"[tiab] OR "baseline and"[tiab] OR "baseline, and"[tiab] OR "dynamic change*"[tiab] OR "time course"[tiab] OR "over time"[tiab] OR "during treatment"[tiab] )

**NT-proBNP**

**NT-proBNP × Anthracycline (PubMed)**

( Anthracyclines[Mesh] OR anthracycline*[tiab] OR doxorubicin[tiab] OR adriamycin[tiab] OR epirubicin[tiab] OR daunorubicin[tiab] OR idarubicin[tiab] OR "liposomal doxorubicin"[tiab] OR "pegylated liposomal doxorubicin"[tiab] ) AND ( Natriuretic Peptide, Brain[Mesh] OR "NT-proBNP"[tiab] OR "NT proBNP"[tiab] OR "NTproBNP"[tiab] OR "N-terminal pro B-type natriuretic peptide"[tiab] OR "N-terminal pro-B-type natriuretic peptide"[tiab] OR "amino-terminal pro-B-type natriuretic peptide"[tiab] ) AND ( Cardiotoxicity[Mesh] OR Heart Failure[Mesh] OR Ventricular Dysfunction, Left[Mesh] OR Cardiomyopathies[Mesh] OR cardiotoxic*[tiab] OR "cardiac toxicity"[tiab] OR "cardiac injury"[tiab] OR "cardiac adverse event*"[tiab] OR "heart failure"[tiab] OR "left ventricular ejection fraction"[tiab] OR LVEF[tiab] OR "systolic dysfunction"[tiab] OR cardiomyopathy[tiab] OR "cardiac dysfunction"[tiab] ) AND ( "Follow-Up Studies"[Mesh] OR "Longitudinal Studies"[Mesh] OR "Time Factors"[Mesh] OR longitudinal[tiab] OR "follow-up"[tiab] OR "follow up"[tiab] OR serial[tiab] OR sequential[tiab] OR monitoring[tiab] OR surveillance[tiab] OR "dynamic change*"[tiab] OR "time course"[tiab] OR "over time"[tiab] OR "during treatment"[tiab] ) AND Humans[Mesh]

**NT-proBNP × HER2 inhibitor (PubMed)**

( "Receptor, ErbB-2"[Mesh] OR "Trastuzumab"[Mesh] OR her2[tiab] OR "HER2"[tiab] OR "HER2-positive"[tiab] OR "HER2 positive"[tiab] OR "HER2-targeted"[tiab] OR "HER2 targeted"[tiab] OR "anti-HER2"[tiab] OR trastuzumab[tiab] OR pertuzumab[tiab] OR lapatinib[tiab] OR neratinib[tiab] OR tucatinib[tiab] OR "ado-trastuzumab emtansine"[tiab] OR "trastuzumab emtansine"[tiab] OR "T-DM1"[tiab] OR "trastuzumab deruxtecan"[tiab] OR "T-DXd"[tiab] ) AND ( "Natriuretic Peptide, Brain"[Mesh] OR "NT-proBNP"[tiab] OR "NT proBNP"[tiab] OR "NTproBNP"[tiab] OR "N-terminal pro B-type natriuretic peptide"[tiab] OR "N-terminal pro-B-type natriuretic peptide"[tiab] OR "amino-terminal pro-B-type natriuretic peptide"[tiab] ) AND ( "Cardiotoxicity"[Mesh] OR "Heart Failure"[Mesh] OR "Ventricular Dysfunction, Left"[Mesh] OR "Cardiomyopathies"[Mesh] OR cardiotoxic*[tiab] OR "cardiac toxicity"[tiab] OR "cardiac injury"[tiab] OR "cardiac adverse event*"[tiab] OR "heart failure"[tiab] OR "left ventricular ejection fraction"[tiab] OR "LVEF"[tiab] OR "systolic dysfunction"[tiab] OR cardiomyopathy[tiab] OR "cardiac dysfunction"[tiab] ) AND ( "Follow-Up Studies"[Mesh] OR "Longitudinal Studies"[Mesh] OR "Time Factors"[Mesh] OR longitudinal[tiab] OR "follow-up"[tiab] OR "follow up"[tiab] OR serial[tiab] OR sequential[tiab] OR monitoring[tiab] OR surveillance[tiab] OR "dynamic change*"[tiab] OR "time course"[tiab] OR "over time"[tiab] OR "during treatment"[tiab] ) AND "Humans"[Mesh]

**NT-proBNP × Immune checkpoint inhibitor (PubMed)**

( "Immune Checkpoint Inhibitors"[Mesh] OR "immune checkpoint inhibitor*"[tiab] OR "immune-checkpoint inhibitor*"[tiab] OR ICI[tiab] OR ICIs[tiab] OR "PD-1 inhibitor*"[tiab] OR "PD-L1 inhibitor*"[tiab] OR "CTLA-4 inhibitor*"[tiab] OR nivolumab[tiab] OR pembrolizumab[tiab] OR cemiplimab[tiab] OR atezolizumab[tiab] OR durvalumab[tiab] OR avelumab[tiab] OR ipilimumab[tiab] OR tremelimumab[tiab] ) AND ( Natriuretic Peptide, Brain[Mesh] OR "NT-proBNP"[tiab] OR "NT proBNP"[tiab] OR "NTproBNP"[tiab] OR "N-terminal pro B-type natriuretic peptide"[tiab] OR "N-terminal pro-B-type natriuretic peptide"[tiab] OR "amino-terminal pro-B-type natriuretic peptide"[tiab] ) AND ( Cardiotoxicity[Mesh] OR Myocarditis[Mesh] OR Heart Failure[Mesh] OR Ventricular Dysfunction, Left[Mesh] OR Cardiomyopathies[Mesh] OR myocarditis[tiab] OR cardiotoxic*[tiab] OR "cardiac toxicity"[tiab] OR "cardiac injury"[tiab] OR "cardiac adverse event*"[tiab] OR "heart failure"[tiab] OR "left ventricular ejection fraction"[tiab] OR LVEF[tiab] OR "systolic dysfunction"[tiab] OR cardiomyopathy[tiab] OR "cardiac dysfunction"[tiab] ) AND ( "Follow-Up Studies"[Mesh] OR "Longitudinal Studies"[Mesh] OR "Time Factors"[Mesh] OR longitudinal[tiab] OR "follow-up"[tiab] OR "follow up"[tiab] OR serial[tiab] OR sequential[tiab] OR monitoring[tiab] OR surveillance[tiab] OR "dynamic change*"[tiab] OR "time course"[tiab] OR "over time"[tiab] OR "during treatment"[tiab] ) AND Humans[Mesh]

**NT-proBNP × VEGF/VEGFR inhibitor (PubMed)**

( "Vascular Endothelial Growth Factor A"[Mesh] OR "Angiogenesis Inhibitors"[Mesh] OR "Bevacizumab"[Mesh] OR bevacizumab[tiab] OR aflibercept[tiab] OR ramucirumab[tiab] OR ranibizumab[tiab] OR "anti-VEGF"[tiab] OR "anti VEGF"[tiab] OR "VEGF inhibitor*"[tiab] OR "VEGF-targeted"[tiab] OR "VEGF targeted"[tiab] OR "VEGFR inhibitor*"[tiab] OR sunitinib[tiab] OR sorafenib[tiab] OR pazopanib[tiab] OR axitinib[tiab] OR cabozantinib[tiab] ) AND ( Natriuretic Peptide, Brain[Mesh] OR "NT-proBNP"[tiab] OR "NT proBNP"[tiab] OR "NTproBNP"[tiab] OR "N-terminal pro B-type natriuretic peptide"[tiab] OR "N-terminal pro-B-type natriuretic peptide"[tiab] OR "amino-terminal pro-B-type natriuretic peptide"[tiab] ) AND ( Cardiotoxicity[Mesh] OR Heart Failure[Mesh] OR Ventricular Dysfunction, Left[Mesh] OR Cardiomyopathies[Mesh] OR cardiotoxic*[tiab] OR "cardiac toxicity"[tiab] OR "cardiac injury"[tiab] OR "cardiac adverse event*"[tiab] OR "heart failure"[tiab] OR "left ventricular ejection fraction"[tiab] OR LVEF[tiab] OR "systolic dysfunction"[tiab] OR cardiomyopathy[tiab] OR "cardiac dysfunction"[tiab] ) AND ( "Follow-Up Studies"[Mesh] OR "Longitudinal Studies"[Mesh] OR "Time Factors"[Mesh] OR longitudinal[tiab] OR "follow-up"[tiab] OR "follow up"[tiab] OR serial[tiab] OR sequential[tiab] OR monitoring[tiab] OR surveillance[tiab] OR "dynamic change*"[tiab] OR "time course"[tiab] OR "over time"[tiab] OR "during treatment"[tiab] ) AND Humans[Mesh]

**BNP**

**BNP × Anthracycline (PubMed)**

( Anthracyclines[Mesh] OR anthracycline*[tiab] OR doxorubicin[tiab] OR adriamycin[tiab] OR epirubicin[tiab] OR daunorubicin[tiab] OR idarubicin[tiab] OR "liposomal doxorubicin"[tiab] OR "pegylated liposomal doxorubicin"[tiab] ) AND ( BNP[tiab] OR "B-type natriuretic peptide"[tiab] OR "B type natriuretic peptide"[tiab] OR "brain natriuretic peptide"[tiab] ) AND ( Cardiotoxicity[Mesh] OR Heart Failure[Mesh] OR Ventricular Dysfunction, Left[Mesh] OR Cardiomyopathies[Mesh] OR cardiotoxic*[tiab] OR "cardiac toxicity"[tiab] OR "cardiac injury"[tiab] OR "cardiac adverse event*"[tiab] OR "heart failure"[tiab] OR "left ventricular ejection fraction"[tiab] OR LVEF[tiab] OR "systolic dysfunction"[tiab] OR cardiomyopathy[tiab] OR "cardiac dysfunction"[tiab] ) AND ( "Follow-Up Studies"[Mesh] OR "Longitudinal Studies"[Mesh] OR "Time Factors"[Mesh] OR longitudinal[tiab] OR "follow-up"[tiab] OR "follow up"[tiab] OR serial[tiab] OR sequential[tiab] OR monitoring[tiab] OR surveillance[tiab] OR "dynamic change*"[tiab] OR "time course"[tiab] OR "over time"[tiab] OR "during treatment"[tiab] ) AND Humans[Mesh]

**BNP × HER2 inhibitor (PubMed)**

( "Receptor, ErbB-2"[Mesh] OR "Trastuzumab"[Mesh] OR her2[tiab] OR "HER2"[tiab] OR "HER2-positive"[tiab] OR "HER2 positive"[tiab] OR "HER2-targeted"[tiab] OR "HER2 targeted"[tiab] OR "anti-HER2"[tiab] OR trastuzumab[tiab] OR pertuzumab[tiab] OR lapatinib[tiab] OR neratinib[tiab] OR tucatinib[tiab] OR "ado-trastuzumab emtansine"[tiab] OR "trastuzumab emtansine"[tiab] OR "T-DM1"[tiab] OR "trastuzumab deruxtecan"[tiab] OR "T-DXd"[tiab] ) AND ( BNP[tiab] OR "B-type natriuretic peptide"[tiab] OR "B type natriuretic peptide"[tiab] OR "brain natriuretic peptide"[tiab] ) AND ( Cardiotoxicity[Mesh] OR Heart Failure[Mesh] OR Ventricular Dysfunction, Left[Mesh] OR Cardiomyopathies[Mesh] OR cardiotoxic*[tiab] OR "cardiac toxicity"[tiab] OR "cardiac injury"[tiab] OR "cardiac adverse event*"[tiab] OR "heart failure"[tiab] OR "left ventricular ejection fraction"[tiab] OR LVEF[tiab] OR "systolic dysfunction"[tiab] OR cardiomyopathy[tiab] OR "cardiac dysfunction"[tiab] ) AND ( "Follow-Up Studies"[Mesh] OR "Longitudinal Studies"[Mesh] OR "Time Factors"[Mesh] OR longitudinal[tiab] OR "follow-up"[tiab] OR "follow up"[tiab] OR serial[tiab] OR sequential[tiab] OR monitoring[tiab] OR surveillance[tiab] OR "dynamic change*"[tiab] OR "time course"[tiab] OR "over time"[tiab] OR "during treatment"[tiab] ) AND Humans[Mesh]

**BNP × Immune checkpoint inhibitor (PubMed)**

( "Immune Checkpoint Inhibitors"[Mesh] OR "immune checkpoint inhibitor*"[tiab] OR "immune-checkpoint inhibitor*"[tiab] OR ICI[tiab] OR ICIs[tiab] OR "PD-1 inhibitor*"[tiab] OR "PD-L1 inhibitor*"[tiab] OR "CTLA-4 inhibitor*"[tiab] OR nivolumab[tiab] OR pembrolizumab[tiab] OR cemiplimab[tiab] OR atezolizumab[tiab] OR durvalumab[tiab] OR avelumab[tiab] OR ipilimumab[tiab] OR tremelimumab[tiab] ) AND ( BNP[tiab] OR "B-type natriuretic peptide"[tiab] OR "B type natriuretic peptide"[tiab] OR "brain natriuretic peptide"[tiab] ) AND ( Cardiotoxicity[Mesh] OR Myocarditis[Mesh] OR Heart Failure[Mesh] OR Ventricular Dysfunction, Left[Mesh] OR Cardiomyopathies[Mesh] OR myocarditis[tiab] OR cardiotoxic*[tiab] OR "cardiac toxicity"[tiab] OR "cardiac injury"[tiab] OR "cardiac adverse event*"[tiab] OR "heart failure"[tiab] OR "left ventricular ejection fraction"[tiab] OR LVEF[tiab] OR "systolic dysfunction"[tiab] OR cardiomyopathy[tiab] OR "cardiac dysfunction"[tiab] ) AND ( "Follow-Up Studies"[Mesh] OR "Longitudinal Studies"[Mesh] OR "Time Factors"[Mesh] OR longitudinal[tiab] OR "follow-up"[tiab] OR "follow up"[tiab] OR serial[tiab] OR sequential[tiab] OR monitoring[tiab] OR surveillance[tiab] OR "dynamic change*"[tiab] OR "time course"[tiab] OR "over time"[tiab] OR "during treatment"[tiab] ) AND Humans[Mesh]

**BNP × VEGF/VEGFR inhibitor (PubMed)**

( "Vascular Endothelial Growth Factor A"[Mesh] OR "Angiogenesis Inhibitors"[Mesh] OR "Bevacizumab"[Mesh] OR bevacizumab[tiab] OR aflibercept[tiab] OR ramucirumab[tiab] OR ranibizumab[tiab] OR "anti-VEGF"[tiab] OR "anti VEGF"[tiab] OR "VEGF inhibitor*"[tiab] OR "VEGF-targeted"[tiab] OR "VEGF targeted"[tiab] OR "VEGFR inhibitor*"[tiab] OR sunitinib[tiab] OR sorafenib[tiab] OR pazopanib[tiab] OR axitinib[tiab] OR cabozantinib[tiab] ) AND ( BNP[tiab] OR "B-type natriuretic peptide"[tiab] OR "B type natriuretic peptide"[tiab] OR "brain natriuretic peptide"[tiab] ) AND ( Cardiotoxicity[Mesh] OR Heart Failure[Mesh] OR Ventricular Dysfunction, Left[Mesh] OR Cardiomyopathies[Mesh] OR cardiotoxic*[tiab] OR "cardiac toxicity"[tiab] OR "cardiac injury"[tiab] OR "cardiac adverse event*"[tiab] OR "heart failure"[tiab] OR "left ventricular ejection fraction"[tiab] OR LVEF[tiab] OR "systolic dysfunction"[tiab] OR cardiomyopathy[tiab] OR "cardiac dysfunction"[tiab] ) AND ( "Follow-Up Studies"[Mesh] OR "Longitudinal Studies"[Mesh] OR "Time Factors"[Mesh] OR longitudinal[tiab] OR "follow-up"[tiab] OR "follow up"[tiab] OR serial[tiab] OR sequential[tiab] OR monitoring[tiab] OR surveillance[tiab] OR "dynamic change*"[tiab] OR "time course"[tiab] OR "over time"[tiab] OR "during treatment"[tiab] ) AND Humans[Mesh]

**sST2**

**sST2 × Anthracycline (PubMed)**

( Anthracyclines[Mesh] OR anthracycline*[tiab] OR doxorubicin[tiab] OR adriamycin[tiab] OR epirubicin[tiab] OR daunorubicin[tiab] OR idarubicin[tiab] OR "liposomal doxorubicin"[tiab] OR "pegylated liposomal doxorubicin"[tiab] ) AND ( ST2 Protein[Mesh] OR ST2[tiab] OR "sST2"[tiab] OR "soluble ST2"[tiab] OR "suppression of tumorigenicity 2"[tiab] OR "IL1RL1"[tiab] OR "interleukin-1 receptor-like 1"[tiab] ) AND ( Cardiotoxicity[Mesh] OR Heart Failure[Mesh] OR Ventricular Dysfunction, Left[Mesh] OR Cardiomyopathies[Mesh] OR cardiotoxic*[tiab] OR "cardiac toxicity"[tiab] OR "cardiac injury"[tiab] OR "cardiac adverse event*"[tiab] OR "heart failure"[tiab] OR "left ventricular ejection fraction"[tiab] OR LVEF[tiab] OR "systolic dysfunction"[tiab] OR cardiomyopathy[tiab] OR "cardiac dysfunction"[tiab] ) AND ( "Follow-Up Studies"[Mesh] OR "Longitudinal Studies"[Mesh] OR "Time Factors"[Mesh] OR longitudinal[tiab] OR "follow-up"[tiab] OR "follow up"[tiab] OR serial[tiab] OR sequential[tiab] OR monitoring[tiab] OR surveillance[tiab] OR "dynamic change*"[tiab] OR "time course"[tiab] OR "over time"[tiab] OR "during treatment"[tiab] ) AND Humans[Mesh]

**sST2 × HER2 inhibitor (PubMed)**

( "Receptor, ErbB-2"[Mesh] OR "Trastuzumab"[Mesh] OR her2[tiab] OR "HER2"[tiab] OR "HER2-positive"[tiab] OR "HER2 positive"[tiab] OR "HER2-targeted"[tiab] OR "HER2 targeted"[tiab] OR "anti-HER2"[tiab] OR trastuzumab[tiab] OR pertuzumab[tiab] OR lapatinib[tiab] OR neratinib[tiab] OR tucatinib[tiab] OR "ado-trastuzumab emtansine"[tiab] OR "trastuzumab emtansine"[tiab] OR "T-DM1"[tiab] OR "trastuzumab deruxtecan"[tiab] OR "T-DXd"[tiab] ) AND ( "ST2 protein"[Mesh] OR "ST2"[tiab] OR "sST2"[tiab] OR "soluble ST2"[tiab] OR "suppression of tumorigenicity 2"[tiab] OR "IL1RL1"[tiab] OR "interleukin-1 receptor-like 1"[tiab] ) AND ( Cardiotoxicity[Mesh] OR Heart Failure[Mesh] OR Ventricular Dysfunction, Left[Mesh] OR Cardiomyopathies[Mesh] OR cardiotoxic*[tiab] OR "cardiac toxicity"[tiab] OR "cardiac injury"[tiab] OR "cardiac adverse event*"[tiab] OR "heart failure"[tiab] OR "left ventricular ejection fraction"[tiab] OR LVEF[tiab] OR "systolic dysfunction"[tiab] OR cardiomyopathy[tiab] OR "cardiac dysfunction"[tiab] ) AND ( "Follow-Up Studies"[Mesh] OR "Longitudinal Studies"[Mesh] OR "Time Factors"[Mesh] OR longitudinal[tiab] OR "follow-up"[tiab] OR "follow up"[tiab] OR serial[tiab] OR sequential[tiab] OR monitoring[tiab] OR surveillance[tiab] OR "dynamic change*"[tiab] OR "time course"[tiab] OR "over time"[tiab] OR "during treatment"[tiab] ) AND Humans[Mesh]

**sST2 × Immune checkpoint inhibitor [evidence-sparse combination] (PubMed)**

( "Immune Checkpoint Inhibitors"[Mesh] OR "immune checkpoint inhibitor*"[tiab] OR "immune-checkpoint inhibitor*"[tiab] OR ICI[tiab] OR ICIs[tiab] OR "PD-1 inhibitor*"[tiab] OR "PD-L1 inhibitor*"[tiab] OR "CTLA-4 inhibitor*"[tiab] OR nivolumab[tiab] OR pembrolizumab[tiab] OR cemiplimab[tiab] OR atezolizumab[tiab] OR durvalumab[tiab] OR avelumab[tiab] OR ipilimumab[tiab] OR tremelimumab[tiab] ) AND ( sST2[tiab] OR "soluble ST2"[tiab] OR ST2[tiab] OR "suppression of tumorigenicity 2"[tiab] OR IL1RL1[tiab] OR "interleukin-1 receptor-like 1"[tiab] ) AND ( Cardiotoxicity[Mesh] OR Myocarditis[Mesh] OR Heart Failure[Mesh] OR Ventricular Dysfunction, Left[Mesh] OR Cardiomyopathies[Mesh] OR cardiotoxic*[tiab] OR myocarditis[tiab] OR "cardiac toxicity"[tiab] OR "cardiac injury"[tiab] OR "cardiac adverse event*"[tiab] OR "heart failure"[tiab] OR "left ventricular ejection fraction"[tiab] OR LVEF[tiab] OR "systolic dysfunction"[tiab] OR cardiomyopathy[tiab] OR "cardiac dysfunction"[tiab] ) AND ( "Follow-Up Studies"[Mesh] OR "Longitudinal Studies"[Mesh] OR "Time Factors"[Mesh] OR longitudinal[tiab] OR "follow-up"[tiab] OR "follow up"[tiab] OR serial[tiab] OR sequential[tiab] OR monitoring[tiab] OR surveillance[tiab] OR "dynamic change*"[tiab] OR "time course"[tiab] OR "over time"[tiab] OR "during treatment"[tiab] ) AND Humans[Mesh]

**sST2 × VEGF/VEGFR inhibitor [evidence-sparse combination] (PubMed)**

( "Vascular Endothelial Growth Factor Inhibitors"[Mesh] OR "Angiogenesis Inhibitors"[Mesh] OR VEGF[tiab] OR VEGF-targeted[tiab] OR anti-VEGF[tiab] OR bevacizumab[tiab] OR ramucirumab[tiab] OR aflibercept[tiab] OR sunitinib[tiab] OR sorafenib[tiab] OR pazopanib[tiab] OR axitinib[tiab] OR lenvatinib[tiab] OR regorafenib[tiab] OR cabozantinib[tiab] ) AND ( sST2[tiab] OR "soluble ST2"[tiab] OR ST2[tiab] OR "suppression of tumorigenicity 2"[tiab] OR IL1RL1[tiab] OR "interleukin-1 receptor-like 1"[tiab] ) AND ( Cardiotoxicity[Mesh] OR Heart Failure[Mesh] OR Ventricular Dysfunction, Left[Mesh] OR Cardiomyopathies[Mesh] OR cardiotoxic*[tiab] OR "cardiac toxicity"[tiab] OR "cardiac injury"[tiab] OR "cardiac adverse event*"[tiab] OR "heart failure"[tiab] OR "left ventricular ejection fraction"[tiab] OR LVEF[tiab] OR "systolic dysfunction"[tiab] OR cardiomyopathy[tiab] OR "cardiac dysfunction"[tiab] ) AND ( "Follow-Up Studies"[Mesh] OR "Longitudinal Studies"[Mesh] OR "Time Factors"[Mesh] OR longitudinal[tiab] OR "follow-up"[tiab] OR "follow up"[tiab] OR serial[tiab] OR sequential[tiab] OR monitoring[tiab] OR surveillance[tiab] OR "dynamic change*"[tiab] OR "time course"[tiab] OR "over time"[tiab] OR "during treatment"[tiab] ) AND Humans[Mesh]

**GDF-15**

**GDF-15 × Anthracycline (PubMed)**

*[Full query string not preserved in the source workbook for this combination; the search used the corresponding treatment-specific template used for the other biomarkers with the GDF-15 biomarker block: "GDF-15"[tiab] OR GDF15[tiab] OR "growth differentiation factor 15"[tiab] OR "growth differentiation factor-15"[tiab] OR MIC-1[tiab] OR NAG-1[tiab]. Eligible extracted records for this combination are documented in Supplementary Table S1.]*

**GDF-15 × HER2 inhibitor (PubMed)**

*[Full query string not preserved in the source workbook for this combination; the search used the corresponding treatment-specific template used for the other biomarkers with the GDF-15 biomarker block: "GDF-15"[tiab] OR GDF15[tiab] OR "growth differentiation factor 15"[tiab] OR "growth differentiation factor-15"[tiab] OR MIC-1[tiab] OR NAG-1[tiab]. Eligible extracted records for this combination are documented in Supplementary Table S1.]*

*[16 of 18 biomarker×treatment PubMed query strings reproduced verbatim; the 2 GDF-15 combinations followed the same template as noted.]*
